# Hemodynamic impact of isobaric levobupivacaine versus hyperbaric bupivacaine for subarachnoid anesthesia in patients aged 65 and older undergoing hip surgery

**DOI:** 10.1186/1471-2253-14-97

**Published:** 2014-10-24

**Authors:** Rosa Herrera, Jose De Andrés, Luis Estañ, Francisco J Morales Olivas, Inocencia Martínez-Mir, Thorsten Steinfeldt

**Affiliations:** Department of Anesthesiology, Critical Care and Pain Management, Consorcio Hospital General Universitario (CHGUV), Valencia, Spain; Department of Surgery, Medical School, University of Valencia, Valencia, Spain; Medical School, University of Valencia, Valencia, Spain; Fundación Hospital General Universitario, Valencia, Spain; Department of Anaesthesiology and Intensive Care Therapy, University Hospital Giessen-Marburg, Faculty of Medicine, Philipps University, Marburg, Germany

**Keywords:** Elderly, Hip fracture, Subarachnoid block, Levobupivacaine, Bupivacaine

## Abstract

**Background:**

The altered hemodynamics, and therefore the arterial hypotension is the most prevalent adverse effect after subarachnoid anesthesia. The objective of the study was to determine the exact role of local anesthetic selection underlying spinal anesthesia-induced hypotension in the elderly patient. We conducted a descriptive, observational pilot study to assess the hemodynamic impact of subarachnoid anesthesia with isobaric levobupivacaine versus hyperbaric bupivacaine for hip fracture surgery.

**Description:**

Hundred twenty ASA status I-IV patients aged 65 and older undergoing hip fracture surgery were enrolled. The primary objective of our study was to compare hemodynamic effects based on systolic blood pressure (SBP) and dyastolic blood pressure (DBP) values, heart rate (HR) and hemoglobin (Hb) and respiratory effects based on partial oxygen saturation (SpO_2_%) values. The secondary objective was to assess potential adverse events with the use of levobupivacaine versus bupivacaine. Assessments were performed preoperatively, at 30 minutes into surgery, at the end of anesthesia and at 48 hours and 6 months after surgery.

Among intraoperative events, the incidence of hypotension was statistically significantly higher (*p* <0.05) in group BUPI (38.3%) compared to group LEVO (13.3%). There was a decrease (*p* <0.05) in systolic blood pressure (SBP) and diastolic blood pressure (DBP) at 30 minutes intraoperatively (19% in group BUPI versus 17% in group LEVO). SpO_2_% increased at 30 minutes after anesthesia onset (1% in group BUPI versus 1.5% in group LEVO). Heart rate (HR) decreased at 30 minutes after anesthesia onset (5% in group BUPI versus 9% in group L). Hemoglobin (Hb) decreased from time of operating room (OR) admission to the end of anesthesia (9.3% in group BUPI versus 12.5% in group LEVO). The incidence of red blood cell (RBC) transfusion was 13.3% in group BUPI versus 31.7% in group LEVO, this difference was statistically significant. Among postoperative events, the incidence of congestive heart failure (CHF) was significantly higher in group BUPI (8,3%). At 6 months after anesthesia, no differences were found.

**Conclusions:**

Given the hemodynamic stability and lower incidence of intraoperative hypotension observed, levobupivacaine could be the agent of choice for subarachnoid anesthesia in elderly patients.

## Background

Hip fractures are common in the elderly population of developed countries. Approximately 30,000 hip fractures occur in Spain every year, at an average age of 80 years [1]. Hospital mortality in Spain due to hip fractures ranges between 4% and 8% [2] and can be as high as 30% at 12 months after hip fracture surgery [3, 4] _._

Perioperative morbidity and mortality can be influenced by both the anesthetic agent [5] and the surgical procedures. Pathophysiological changes associated with aging, significant comorbidities, treatment with multiple medications and a reduced functional reserve render the elderly more vulnerable to the pharmacological effects of drugs in general and particularly to local and general anesthetics [6]. Racemic bupivacaine is considered the long-acting local anesthetic of choice in several regional anesthetic procedures [7–9], especially for subarachnoid administration. Levobupivacaine is the S-enantiomer of racemic bupivacaine. Clinical studies have shown that bupivacaine and levobupivacaine are equally effective [5, 10], however, levobupivacaine has lower affinity for sodium channels in the heart [11], and therefore it is less frequently associated with cardiovascular (CV) events.

A recent systematic review [12] suggests that neuraxial (subarachnoid and epidural) regional techniques reduce to 1/3 the incidence of postoperative myocardial infarction; these authors recommend preventing hypotension associated with spinal blocks, hypoxia and anemia, as these may lead to the occurrence of perioperative adverse events [13, 14].

The **primary objective** of our study was to compare hemodynamic effects based on systolic blood pressure (SBP) and dyastolic blood pressure (DBP) values, heart rate (HR) and hemoglobin (Hb) and respiratory effects based on partial oxygen saturation (SpO_2_%) values. The **secondary objective** of the study was to assess potential adverse events with the use of isobaric levobupivacaine versus hyperbaric bupivacaine, associated with both the surgical and the anesthetic procedures, and death.

There is little evidence comparing the use of levobupivacaine [10] versus bupivacaine, either in clinical practice [15, 16] or in studies assessing the safety of one versus the other [17–21]. Assessments were conducted preoperatively, at 30 minutes after anesthesia, at the end of anesthesia (when the patient could be transferred to the post surgical recovery unit), and at 48 hours and 6 months postoperatively. The ideal subarachnoid block for management of aged 65 and older undergoing hip surgery remains elusive, especially in respect of dosing and local anesthetic selection. To explore these issues, we compare two differing local anesthetics (LA) formulations. Comparative evidence on the effectiveness of the two LA was obtained through a structured database.

## Construction and content

This was a descriptive, observational pilot study to assess the hemodynamic effects of subarachnoid anesthesia with isobaric levobupivacaine versus hyperbaric bupivacaine in 120 ASA status I-IV patients aged 65 and older undergoing hip fracture surgery at Consorcio Hospital General Universitario (CHGUV), Valencia, Spain. Assessments were conducted preoperatively, at 30 minutes of the anesthesia, at the end of anesthesia and at 48 hours and 6 months postoperatively.

Patients were divided into two groups based on the type of anesthetic solution used: group LEVO included patients with 0.5% isobaric levobupivacaine (Chirocane™, Abbott) plus fentanyl 50 μg/mL (solution A), and group BUPI included patients with 0.5% hyperbaric bupivacaine (Braun, Rubí, Spain) plus fentanyl 50 μg/mL (solution B).

A total of 120 patients were included (60 patients in the isobaric levobupivacaine group and 60 in the hyperbaric bupivacaine group).

Inclusion criteria were as follows: males and females aged 65 or older diagnosed with a hip fracture and treated with intrathecal anesthesia with levobupivacaine plus fentanyl or bupivacaine plus fentanyl (hyperbaric bupivacaine and isobaric levobupivacaine dosages ranged between 5 mg and 15 mg, and the dose range for fentanyl was 10 μg to 20 μg); patients with or without stable cardiovascular, respiratory, renal and/or endocrine disease classified as I-IV according to the American Society of Anesthesiologists (ASA); body weight >40 kg; height >140 cm; body mass index (BMI) <50 kg/m^2^.

We excluded those patients who underwent spinal anesthesia with general anesthesia that required by technical failure or prolonged surgical time.

Before the study was initiated, approval was obtained from the Research Commission and the Clinical Research Ethics Committee at the Department of Health of Valencia, Hospital General (no. 538, no. 1) and the project was reviewed by the Spanish Regulatory Drug Agency (AEMPS) and classified as a ‘post-marketing, non-prospective follow-up study’, protocol number RHC-LEV-2012-01.

The study was conducted in accordance with the principles laid out in the Declaration of Helsinki and the applicable law and regulations governing personal data protection and rights and responsibilities regarding information and documentation in healthcare.

The standard subarachnoid technique was used, with the patient placed in the lateral position with the affected limb raised or lowered as chosen by the designated anesthesiologist. After sterilizing the anesthetic field, local infiltration was performed using 2% lidocaine. Both solution A or solution B of the anesthetic were administered by the anesthesiologist (from the Anesthesiology department allocated to the operating room (OR) according to the hospital’s organizational chart) aseptically in the subarachnoid space using a Whitacre 25 G or 27 G needle. Puncture was performed using the midline or paramedian approach in intervertebral spaces L2-L3, L3-L4 or L4-L5, with the bevel in the cephalic or caudal direction. After confirmation of clear cerebrospinal fluid (CSF) efflux, solution A (*n* =60) or solution B (*n* =60) was administered. The solution was injected with or without prior aspiration of CSF.

Once the subarachnoid puncture was completed, patients were placed in the supine position and urinary catheterization was performed. Patients were moved off the bed onto the surgical table, where they were positioned for surgery in the lateral or supine position according to the type of fracture and the fixation material to be implanted.

Data were collected by specially trained personnel from the patients’ hospital records in the CHGUV archive, consecutively and retrospectively between January 2010 and November 2011. Personal confidential data items for this database study were processed under government transparency guidance and managed in line with statistics authority guidance on the handling of small numbers to prevent the identification of individuals. To make sure personal data were kept confidential, the sample was anonymized using a double code list. Each patient was assigned a unique two- or three-digit number (01, 02…, 60…, 120), from 01 to 120.

The following data were collected:

Socio-demographic variables were collected: patient data, including unique code number (consecutive study number), gender, age (years), body weight (kg), height (cm), and BMI (kg/m^2^); presence of CV, respiratory, neurological, hepatic/renal or endocrine/metabolic disease, history of anti-platelet or anticoagulant agent use prior to surgery; premedication (midazolam, and/or fentanyl, and/or ketamine); type of hip fracture and hospital stay in days.

Before initiation of surgery, anesthesiologists checked sensory and motor blocks. The level of sensory block was assessed using the pinprick test (1 = hypoalgesia; 2 = analgesia; 3 = analgesia plus hypoesthesia; and 4 = anesthesia) using a 22 G blunt hypodermal needle. The level of motor block was assessed using the modified Bromage scale (0 = no motor block, able to flex hips, knees and ankles; 1 = just able to flex knees, unable to extend legs; 2 = able to move ankles, unable to flex knees; 3 = unable to flex ankles, knees or hips, complete motor block).

Anesthetic and surgical technique variables were collected, such as level of puncture (L2-L3, L3-L4, or L4-L5); volume of local anesthetic (mL) and fentanyl (μg) administered, and type of surgical implant; and surgical times and anesthetic times in minutes.

Non invasive hemodynamic monitoring placing six electrocardiographic leads, heart rate (HR) measured in beats per minute (bpm), the oxygen saturation in % expressed through a pulse oximeter placed on the index finger (SpO_2_%) and blood pressures systolic and diastolic measured in mmHg, haemoglobin in g/dL.

Information was collected on potential adverse events during the intraoperative period. Hypotension and bradycardia were defined as a reduction from baseline by >20% in mean arterial pressure (MAP) and HR, respectively. Adverse events included CV and respiratory events, such as venous gas embolism, deep vein thrombosis (DVT), acute myocardial infarction (AMI), stroke, congestive heart failure (CHF), pneumonia or death, and other events such as acute renal failure (ARF) and vomiting. Events associated with the surgical procedure included red blood cells transfusion (RBC), plasma transfusion (PT), nerve injury (NI), femur fracture; (FF) events associated with the anesthetic procedure included paresthesia, bloody puncture, and others.

The assessment performed at 48 hours postoperatively included the following adverse events: DVT, AMI, stroke, CHF, pneumonia and death; and others such as ARF, urinary tract infection, and vomiting. Events associated with the surgical procedure included RBC transfusion (anesthesiologist’ s choice), plasma transfusion, neurological deficits and surgical site infection.

Potential adverse events occurring at 6 months postoperatively included CV/respiratory events, such as exacerbation of CV disease, exacerbation of respiratory disease, exacerbation of kidney disease, and death. The potential adverse events associated with the surgical procedure were neurological deficits and those associated with the anesthetic procedure were metameric dysesthesia, low back pain, and others.

With a sample size of 120 patients (60 in each group), the study had 85% statistical power to detect minimal differences between groups of 20 mmHg in SBP and DBP, 20 bpm in HR, 5 g Hb, and 2 percentage points in partial oxygen saturation.

A specific case report form was developed for this study and the data were transferred to the SPSS 15.0 software for Windows. The information in the database was checked for quality to avoid inconsistencies and duplicated or inaccurate data.

Descriptive statistics were used and the arithmetic mean and the standard deviation were determined for each variable. Comparisons between groups were performed using the Friedman test. The Mann-Whitney test was used to assess the groups for homogeneity. Pearson’s chi-squared test and Fisher’s exact test were used to assess associations between qualitative variables. Significance was set at *p* <0.05.

Both patient groups had similar socio-demographic characteristics and comorbidities. The most frequent comorbidities were CV and respiratory diseases in both groups. Midazolam, fentanyl and/or ketamine were used for sedation in 76% of all patients (Table 1).

**Table 1:** **Characteristics of patients at baseline**

A. Socio-demographic variables expressed as mean (SD)								
	**N**	**Minimum**	**Maximum**	**Mean**	**Standard deviation**			
**AGE (years)**	119	60	100	84	7.15			
**WEIGHT (kg)**	103	40	120	65	13.02			
**SIZE (cm)**	72	140	181	160	7.92			
**BMI (kg/m** ^**2**^ **)**	71	12	31	20	3.63			
**B. Socio-demographic variables expressed in percentage (%)**								
		**Frequency**	**Percentage**					
**VALID**	**Man**	25	21					
	**Woman**	95	80					
	**Total**	120	100					
**C. Variables related to the main objective**								
	**BUPI (N =60)**				**LEVO (N =60)**			
	**Mean**	**Standard deviation**	**Minimum**	**Maximum**	**Mean**	**Standard deviation**	**Minimum**	**Maximum**
**AGE (years)**	83	7.24	60	96	85	6.92	66	100
**WEIGHT (kg)**	66	14.78	44	120	64	11.32	40	90
**SIZE (cm)**	159	7.83	140	181	160	8.08	140	175
**BMI (kg/m** ^**2**^ **)**	20	3.57	14	27	20	3.73	12	31
**SBP (mmHg)**	143	19.50	110	190	156	28.33	100	230
**DBP (mmHg)**	82	13.74	60	120	79	20.04	50	175
**HR ((bpm)**	85	17.84	60	150	89	21.27	50	170
**Hb (g/dl)**	12	1.62	8	16	12	1.61	8	18
**SpO**_**2**_ **(%)**	96	3.66	85	100	95	2.75	88	99
**D. Clinical variables**								
			**BUPI (N =60)**		**LEVO (N =60)**			
			**N**	**%**	**N**	**%**		
	**GENDER**	**Man**	11	18%	14	23%		
		**Woman**	49	82%	46	77%		
	**BASIC PATHOLOGY**	**RESPIRATORY**	13	22%	8	13%		
		**VASCULAR**	44	73%	39	65%		
		**NEUROLOGICAL**	23	38%	24	40%		
		**CARDIAC**	21	35%	25	42%		
		**HEPATIC/RENAL**	7	12%	10	17%		
		**ENDOCRINE/METABOLIC**	31	52%	30	50%		
		**HISTORY OF ANTI-PLATELET OR ANTICOAGULANT AGENT**	25	42%	26	43%		
**USE PRIOR TO SURGERY: PREMEDICATION**			40	67%	51	85%		
	**TYPE OF HIP FRACTURE**	**Head neck fracture**	0	0%	0	0%		
		**Subcapital fracture**	21	35%	15	25%		
		**Transcervical fracture**	0	0%	2	3%		
		**Basicervical fracture**	6	10%	8	13%		
		**Pertrochanteric fracture**	28	47%	30	50%		
		**Subtrochanteric fracture**	4	7%	5	8%		
**DURING SURGERY**	**OSTEOSYNTHESIS IMPLANT TYPE**	**Osteosynthesis (OS)**	4	7%	2	3%		
		**Partial hip prosthesis (PHP)**	17	28%	17	28%		
		**Total hip prosthesis (THP)**	4	7%	2	3%		
		**Arthroplasty (ARP)**	0	0%	0	0%		
		**Dynamic Hip System plates (DHS)**	28	47%	25	42%		
		**GAMMA plates (GAMMA)**	8	13%	8	13%		
		**Condylar Nail-Plate s (DCS)**	0	0%	0	0%		
		**Others (Oth)**	0	0%	5	8%		
	**SPINAL LEVEL**	**L2-L3**	13	22%	10	17%		
		**L3-L4**	35	58%	31	52%		
		**L4-L5**	12	20%	19	32%		
	**ANESTHESIC VOLUME**	**1 ml**	4	7%	18	30%		
		**1.2 ml**	10	17%	36	60%		
		**1.3-1.5 ml**	32	53%	5	8%		
		**>1.5 ml**	14	23%	1	2%		
	**DOSE OF FENTANYL**	**5**	1	1.7%	0	0%		
		**10**	52	87%	56	93%		
		**15**	3	5%	0	0%		

BUPI = hyperbaric bupivacaine; LEVO = isobaric levobupivacaine; BMI = body mass index; SBP = systolic blood pressure, DBP = diastolic blood pressure; HR = heart rate; Hb = haemoglobin; SpO_2_ = partial oxygen saturation; Osteosynthesis (OS); Partial Hip Prosthesis (PHP); Total Hip Prosthesis (THP); Arthroplasty (ARP).

Dynamic Hip System plates (DHS); GAMMA plates (GAMMA); Condylar Nail-Plates (DCS); Others (Oth).

The levels of sensory and motor block were not provided in the study because they were generally not recorded in the chart of anesthesia.

**Figure 1:**
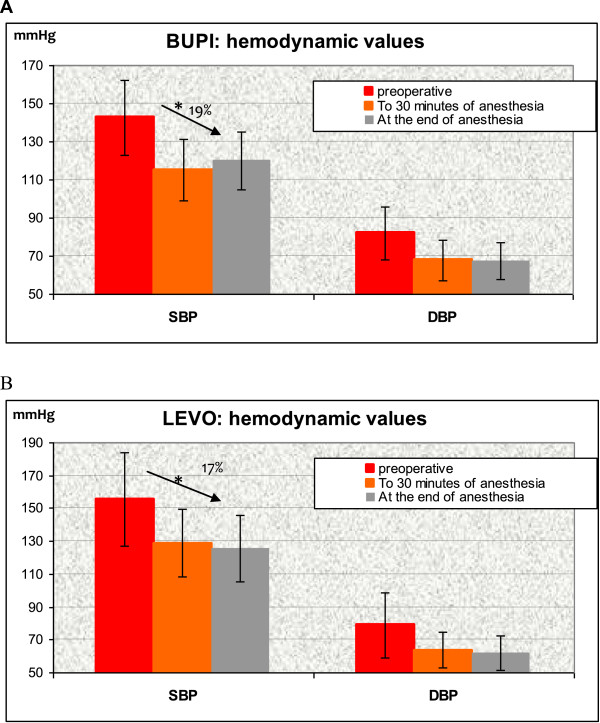
**Effect of bupivacaine (BUPI) (A) and of levobupivacaine (LEVO) (B) on systolic blood pressure (SBP) and diastolic blood pressure (DBP) to the entry in OR admission, at 30 minutes of anesthesia and end of anesthesia.** Significant difference in SBP change at 30 minutes after anesthesia onset (*p* <0.05); BUPI = hyperbaric bupivacaine; LEVO = isobaric levobupivacaine; OR, operating room; SBP = systolic blood pressure; DBP = diastolic blood pressure.

In both groups there is a percentage decrease in SBP (mean preoperative SBP 156 ± 28 mmHg for LEVO and 143 ± 19 mmHg for BUPI) and DBP (mean preoperative DBP 79 ± 20 mmHg for LEVO and 82 ± 14 mmHg for BUPI) after 30 minutes of anesthesia (p <0.05), LEVO 17% and 19% with BUPI. In general, no significant variation appears after the first half hour, however, the BUPI, slightly increases the SBP (mean intraoperative SBP 129 ± 20 mmHg for LEVO and 115 ± 16 mmHg for BUPI and mean SBP at the end of anesthesia 125 ± 20 mmHg for LEVO and 120 ± 15 mmHg for BUPI) (Figure 1).

**Figure 2:**
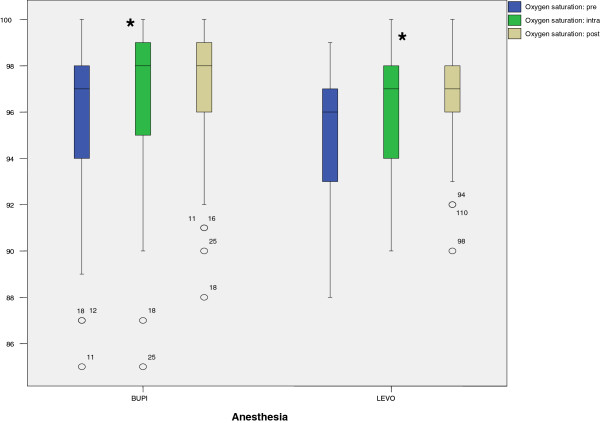
**SpO**_**2**_ **values according to anesthetic time point and anesthetic use (BUPI or LEVO).** BUPI = hyperbaric bupivacaine; LEVO = isobaric levobupivacaine; Oxygen saturation: pre = partial oxygen saturation at operating room (OR) admission; Oxygen saturation: intra = partial oxygen saturation at 30 minutes after onset of anesthesia; Oxygen saturation: post = partial oxygen saturation at the end of anesthesia; SpO_2_% = partial oxygen saturation. ^★^p <0.05 from preoperative values; 0 = atypical value (the numbers next to atypical values refer to case number in the database and not the value of the SpO_2_).

SpO_2_ values at 30 minutes after induction (95% BUPI, 96% LEVO) were significantly increased in percent (p <0.05) as compared to preoperative values (96% BUPI, 95% LEVO). In both groups SpO_2_ increased at 30 minutes after anesthesia of 1% with BUPI and 1.5% with LEVO. There were no differences in these values assessed at 30 minutes after induction of anesthesia versus at the end of anesthesia (97% BUPI, 97% LEVO) (Figure 2).

**Figure 3:**
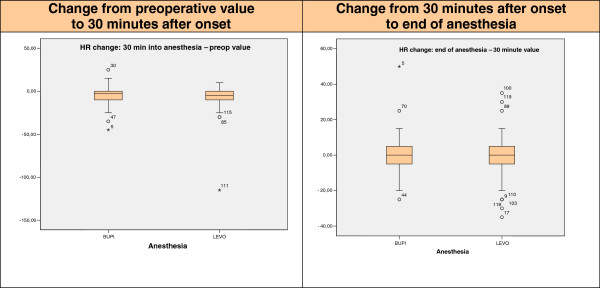
**Heart rate (HR) values according to anesthesia time point.** BUPI = hyperbaric bupivacaine; LEVO = isobaric levobupivacaine; 0 = atypical value (the numbers next to atypical values refer to case number in the database and not the value of the HR); ^★^extreme value.

In both groups there was a percentage decrease of HR at 30 minutes of anesthesia (81 bpm BUPI, 81 bpm LEVO), 5% with BUPI and 9% with LEVO (preoperative values 85 bpm BUPI, 89 bpm LEVO). In general, no significant variation appears after 30 minutes of anesthesia (Figure 3).

**Figure 4:**
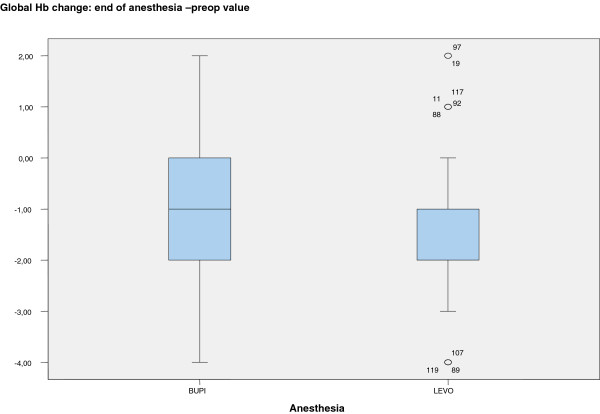
**Hemoglobin values according to local anesthetic (BUPI or LEVO) at end of anesthesia versus OR admission.** BUPI = hyperbaric bupivacaine; LEVO = isobaric levobupivacaine, OR, operating room; 0 = atypical value (the numbers next to atypical values refer to case number in the database and not the value of the Hb).

A significant percentage decrease in Hb occurred with both anesthetics from preoperative values (12 g/dL BUPI, 12 g/dL LEVO) to the end of surgery (11 g/dL BUPI, 10 g/dL LEVO). The mean percentage decrease was 9% with bupivacaine and 12% with levobupivacaine; however, there were no significant differences between both values (Figure 4).

We had not recorded either the eventual use of vasopressors or intraoperative fluid therapy because they were not properly recorded in the charts of anesthesia.

Adverse events assessed intraoperatively, at 48 hours and at 6 months postoperatively are shown in Table 2. Among intraoperative events, the incidence of hypotension was significantly higher (p <0.05) in group BUPI (38%) compared to group LEVO (13%). The incidence of RBC transfusion was 13% in group BUPI versus 32% in group LEVO. These differences were statistically significant. Among postoperative events, the rate of CHF was significantly higher in group BUPI (8%) and was not recorded in the group LEVO. At 6 months after anesthesia, no differences were found in adverse events according to the local anesthetic agent used.

**Table 2:** **Adverse events with bupivacaine and levobupivacaine**

A. Intraoperative adverse events						
	**Total (N =120)**		**BUPI (N =60)**		**LEVO (N =60)**	
	**N**	**%**	**N**	**%**	**N**	**%**
**Stroke**	1	1%	0	0%	1	2%
**Hypotension**	31	26%	3	38%^**★**^	8	13%
**Vomiting**	1	1%	1	2%	0	0%
**RBC transfusion**	27	22%	8	13%	19	32%^**★**^
**B. Adverse events at 48 hours after surgery**						
	**Total (N =120)**		**BUPI (N =60)**		**LEVO (N =60)**	
	**N**	**%**	**N**	**%**		
**Pneumonia**	1	1%	1	2%	0	0%
**CHF**	5	4%	5	8%^**★**^	0	0%
**RF**	4	3%	2	3%	2	3%
**RBC**						
**Transfusion**	34	28%	16	26%	18	30%
**C. Adverse events at 6 months postoperatively**						
	**Total (N =120)**		**BUPI (N =60)**		**LEVO (N =60)**	
	**N**	**%**	**N**	**%**	**N**	**%**
**Exacerbation of kidney disease**	3	2%	2	3	1	2%
**Death**	8	7%	5	8%	3	5%
**Exacerbation of cardio-vascular disease**	5	4%	4	7%	1	2%
**Exacerbation of pulmonary disease**	4	3%	2	3%	2	3%

BUPI = hyperbaric bupivacaine; LEVO = isobaric levobupivacaine; RBC = red blood cells; ARF = acute renal failure; B = hyperbaric bupivacaine; CHF = congestive heart failure; RF = renal failure; ^★^Statistically significant difference (*p* <0.05).

Puncture was performed at L3-L4 in 55% of all patients (58% in group BUPI versus 52% in group LEVO). Subarachnoid block was undertaken using a mean local anesthetic volume of 1.3 ± 0.2 mL (range 2.4-1) and a mean dose of 11 ± 3 μg fentanyl (range 5-20). A higher volume of bupivacaine was used (1.4 mL) compared to levobupivacaine (1.2 mL). In group BUPI, 53% of patients received 1.3 to 1.5 mL, whereas 60% of patients in group LEVO received 1.2 mL. Significant differences were found in the volume of the anesthetic solution but not in the dose of fentanyl: 90% of patients (*n* =108) received 10 μg fentanyl. In group BUPI, patients received a mean dose of 11 ± 2.8 μg fentanyl and group LEVO, patients received a mean dose of 11 ± 2.5 μg fentanyl.

Surgical and anesthetic times were also assessed. Mean surgical time was 58 ± 28 minutes (range 195-25): 61 ± 27 minutes in group BUPI (range 195-30) versus 55 ± 28 minutes in group LEVO (range 160-25). Mean anesthetic time was 92 ± 28 minutes (range 225-45): 97 ± 32 in group BUPI (range 225-45) versus 88 ± 24 in group LEVO (range 150-45).

Mean hospital stay was 8 days (range 20-3) with no significant differences between the groups.

## Utility and discussion

The relative contribution of anaesthesia to outcome after hip fracture repair remains uncertain, but experience across medicine suggests that better outcomes are associated with standardisation of practice. A key objective of the current database has been to present our practice – so as to develop a consensus regarding best practice in hemodynamic impact of subarachnoid LA administration in elderly population with hip fracture. These results will help individual anaesthetists and departments of anaesthesia to improve the management of this sensible population.

Research in the complex, heterogeneous hip fracture population is difficult, making it difficult to formulate strong conclusions. The National Hip Fracture Database [22] is one example of just such a database. Launched in 2007, it has collected data on over 200 000 hip fracture patients, and currently collects data from 95% of all hip fracture patients presenting to each of the 188 hospitals in England, Wales and Northern Ireland that are eligible for inclusion. However, there are some striking findings, most notably the high prevalence of intraoperative hypotension, the limited use of nerve blockade in addition to spinal and/or general anaesthesia and the wide inter-hospital variation in type of anaesthesia administered.

White et al in their database [23] analysed 65 535 patient record sets to determine differences in outcome. Type of anaesthesia was recorded in 59 191 (90%) patients. There was no significant difference in either cumulative five-day (2.8% vs 2.8%, p =0.991) or 30-day (7.0% vs 7.5%, p =0.053) mortality between 30 130 patients receiving general anaesthesia and 22 999 patients receiving spinal anaesthesia. If these data are accurate, research should focus on how to make both types of anaesthesia safer, and therefore future research should focus on differences between general and spinal anaesthesia. These could include more anaesthesia-sensitive outcomes, such as hypotension, pain, postoperative confusion, respiratory infection and mobilisation.

In this sense the main utility of our paper is to assess the hemodynamic impact of levobupivacaine and hyperbaric bupivacaine in patients with 65 or more years with hip fracture during surgery, at 48 hours and at 6 months after surgery. During the intra-operative period was detected a percentage decrease in SBP and DBP after 30 minutes of anesthesia in both groups. This could be explained partly by the baricity of the local anesthetic. Hyperbaric bupivacaine is characterized by earlier onset of action, greater cephalic distribution and greater initial reduction of SBP and DBP. Furthermore, the dosages of the local anesthetics could have influenced the results as well, as the volume of bupivacaine injected was higher than that of levobupivacaine (1.4 mL versus 1.16 mL), which could account for the differences seen at the end of anesthesia.

In these series, among the intraoperative adverse events collected, there was a three times higher incidence of hypotension in group BUPI and two and a half higher incidence of RBC transfusion in group LEVO. In the group treated with bupivacaine, we used more local anesthetic and its onset of action was earlier. This could have favored more hypotension events. As for the transfusions of blood products, Wood et al [19] observed a very slight change in Hb concentrations in patients who received low dosages of local subarachnoid anesthetics compared to higher dosages. These authors suggest that this could be due to less hemodilution as a result of less fluid administration in the group treated with lower dosages. This avoided the use of vasopressors and reduced the administration of intravenous fluid. It is possible that the trend to reduce the administration of fluid may have favored hemoconcentration and therefore hematological loss would have been greater. However, we have not recorded either the eventual use of vasopressors or intraoperative fluid therapy.

At 48 hours after surgery, the incidence rate of heart failure was 8.3% in group BUPI versus no cases in group LEVO. This was possibly related to the therapeutic procedures to counteract hypotension —such as intravenous administration of fluid and vasopressor drugs— and to anemia, leading to interstitial edema and acute pulmonary edema.

At 6 months postoperatively, the number of adverse events was higher in group BUPI than in group LEVO, but the difference was not statistically significant. Death occurred in 6.7% of all patients who underwent surgery, regardless of gender (8.3% in group BUPI versus 5% in group LEVO). These events could be related to the higher prevalence rates of intraoperative hypotension in group BUPI, CHF at 48 hours postoperatively and possibly secondary ischemia involving target organs such as the heart and the kidneys.

In elderly patients with chronic coexistent diseases, episodes of hypotension and operative bleeding may promote the occurrence of intra- and post-operative adverse events, particularly ischemia and anemia [5, 14, 20], which involve a high risk for secondary complications such as AMI, acute and/or chronic renal failure, or death, among others.

Several authors have reported low prevalence rates of intraoperative hypotension with low dosages of subarachnoid bupivacaine and levobupivacaine for hip fracture surgery [10, 20]. The mechanism of this undesirable event remains uncertain; it may be related to lower cephalic diffusion of the local anesthetic and the consequent lower reduction of systemic vascular resistances. Levobupivacaine has been shown to result in greater vasoconstriction at all concentrations compared to racemic bupivacaine [24, 25]. That would explain the lower incidence of hemodynamic effects compared to bupivacaine, which causes vasodilation (leading to arterial hypotension and bradycardia). The prevalence of non-failed subarachnoid block with lower doses of up to 4 mg of anesthetic has been reported in the literature [26] and may be a consequence of the pathophysiological changes occurring with aging and the concurrent diseases in these patients.

Studies can be found in the literature for urological and lower abdominal surgery comparing these two local anesthetics using the subarachnoid approach. However, there are few specific studies in hip surgery, particularly in hip fracture surgery [27, 28].

Wood et al [19] followed a series of 1,131 patients who underwent proximal femur fracture surgery and were treated with 0.5% hyperbaric bupivacaine, observing lower prevalence rates of intraoperative hypotension in the group treated with <1.5 mL. Fattorini et al [15] compared, in a series of only 60 patients undergoing major orthopedic surgery, 3 mL of 0.5% levobupivacaine versus 3 mL of 0.5% bupivacaine for major lower limb surgery and observed a slight decrease in MAP and HR, but no significant differences between the groups.

There were a number of limitations to our study. Collection of data in the anesthetic sheet was not accurate, especially because SBP and DBP values were not collected invasively and because readings of these values could have been influenced subjectively by the investigators. Preoperative Hb values were collected for all cases; however, intra- and postoperative Hb values were missing for some.

We agree with Benes et al that it is essential to ascertain the patient’s blood volume status to determine the appropriate strategy for hemodynamic management [29]. A number of ‘static’ markers had been typically used (central venous pressure (CVP), pulmonary capillary wedge pressure (PCWP)), but their efficacy has been questioned in recent studies [29, 30]. A novel approach, based on some physiological phenomena or new technologies, is now taking hold, supported by new insights in physiology and technology. These are the so-called ‘dynamic’ markers, which can be assessed using electronic devices (such as FloTrac/Vigileo).

## Conclusions

We conclude from our study that subarachnoid administration of low-dose 0.5% levobupivacaine (mean volume of 1.2 mL) plus fentanyl in elderly patients undergoing hip fracture surgery was as safe as the administration of low-dose hyperbaric bupivacaine (mean volume ranging between 1.3 mL and 1.5 mL) plus fentanyl. Our results, especially regarding intra- and postoperative events, suggest that subarachnoid low-dose isobaric levobupivacaine was safer and should be used instead of hyperbaric bupivacaine in elderly patients undergoing surgical hip fracture repair.

## Availability and requirements

All data and related metadata underlying the findings reported in this manuscript has been deposited in the department of Anesthesia in General University Hospital, in addition provided as part of the submitted article. Authors also ensure that the software will be available for a full two years following publication, making materials, data and associated protocols promptly available to readers without undue qualifications.
